# Advances in Molecular Characterization and Targeted Therapy in Dermatofibrosarcoma Protuberans

**DOI:** 10.1155/2011/959132

**Published:** 2011-03-30

**Authors:** Piotr Rutkowski, Agnieszka Wozniak, Tomasz Switaj

**Affiliations:** ^1^Department of Soft Tissue/Bone Sarcoma and Melanoma, Maria Sklodowska-Curie Memorial Cancer Center and Institute of Oncology, Roentgena 5, 02-781 Warsaw, Poland; ^2^Laboratory of Experimental Oncology, Department of General Medical Oncology, Catholic University Leuven and University Hospitals Leuven, 3000 Leuven, Belgium

## Abstract

The molecular pathogenesis of dermatofibrosarcoma protuberans (DFSP) involves distinctive rearrangement of chromosomes 17 and 22 leading to formation of the *COL1A1-PDGFB* fusion gene. The knowledge of molecular events underlying development of DFSP resulted in the implementation of targeted therapy with imatinib—a tyrosine kinase inhibitor (TKI), to the clinical practice. The striking efficacy of imatinib in advanced cases of DFSP has been demonstrated in a few clinical trials. Thus, imatinib is currently considered the gold standard in the treatment of inoperable and/or metastatic and/or recurrent cases of DFSP. Therapy with imatinib may potentially facilitate resection or decrease possible disfigurement related to radical surgical procedure. Following partial response on imatinib significant percentage of patients may be rendered free of the disease by surgery of the residual tumor.

## 1. Introduction

Dermatofibrosarcoma protuberans (DFSP) is a rare cutaneous-origin sarcoma with usually indolent growth (over years) and low metastatic potential. Regional/distant metastases probability is less than 5% [1, 2]. Metastases develop more commonly in DFSP-containing areas of high-grade fibrosarcoma—fibrosarcomatous-DFSP (DFSP-FS) [3–6], which is characterized by more aggressive course. If distant metastases occur they are often restricted to lungs, and less commonly to lymph nodes. The standard treatment of the localized disease is radical, wide local excision. It is recommended that margins of the surgical excision should exceed 2-3 cm [1, 7]. This procedure often requires application of reconstructive techniques and may result in cosmetic disfigurement or functional impairment. Unfortunately, the microscopically infiltrating pattern of tumor growth might lead to high rates of unexpected positive margins. Local recurrences may occur late, and they have been reported within the range of 24–90% [1, 3, 8–14]. Nevertheless, several reports provided data demonstrating lower frequency of recurrence rate [15–17]. Recurrent disease is more challenging surgically, due to tumor fixation to deeper structures. Microscopic infiltrations spreading from the tumor might also lead to high probability of unexpected nonradical resection. There is only limited experience with Mohs micrographic surgery in the treatment of localized DFSP [18–20].

## 2. Molecular Pathogenesis

**Figure 1:**
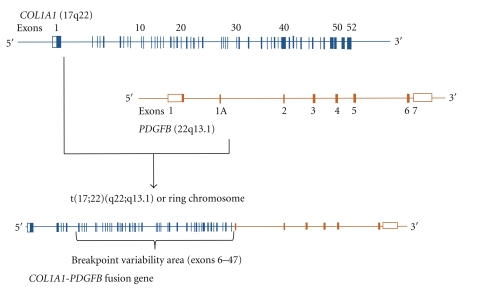
Schematic presentation of the *COL1A1/PDGFB* fusion gene formation.

DFSP is characterized by the presence of distinctive, reciprocal rearrangement of chromosomes 17 and 22 in the form of translocation t(17;22)(q22;q13) or supernumerary ring chromosomes containing material from chromosomal regions 17q22 and 22q 13 [21–31]. The rearrangement leads to the fusion of alpha chain type a (*COL1A1) *localized on 17q22 to the platelet-derived growth factor beta (*PDGFB) *localized on 22q13 (Figure 1) [32].

The *PDGFB* gene product is a growth factor that serves as a ligand for the transmembrane receptor kinase PDGFRB. The formation of *COL1A1-PDGFB *fusion gene results in the constitutional upregulation of *PDGFB* expression, leading to continuous autocrine activation of PDGF receptor B (PDGFRB) and as a consequence to propagation of the mitotic signal by formation of an autocrine and paracrine loops [33–35]. Greco et al. [36, 37] provided evidence, that transfection with *COL1A1-PDGFB *fusion gene could transform NIH3T3 cells. Furthermore, it was shown that by using suramin, a compound known to interfere with PDGF-PDGFR ligand-receptor interaction, the *COL1A1-PDGFB* transformed phenotype in NIH3T3 cells can be reversed [36].

Interestingly the presence of the specific *COL1A1-PDGFB* fusion transcript was also identified in giant cell fibroblastoma (GCF) that is a histologic variant of DFSP. GCF primarily affects children so it is also called the juvenile form of DFSP [38–41]. In DFSP-FS increased copy numbers of *COL1A1-PDGFB* fusion gene were observed suggesting a possible oncogenic mechanism of the clonal evolution from DFSP into DFSP-FS [42]. 

**Figure 2:**
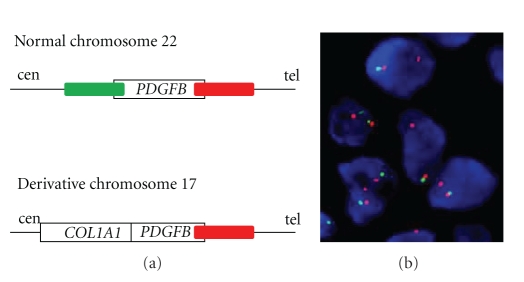
*PDGFB *break-apart FISH in interphase nuclei from DFSP. (a) Schematic localization of FISH probes; (b) *PDGFB *rearrangement detected by FISH, evidenced by one copy (red probe) of the telomeric *PDGFB *signal in tumor cells (courtesy of Professor M. Debiec-Rychter).

Although there is no need for molecular confirmation of the diagnosis in the majority of DFSP cases, the detection of the chromosomal 17;22 rearrangements or the *COL1A1-PDGFB *fusion is a valuable diagnostic tool for differential diagnosis of atypical, metastatic DFSP or DFSP-FS. Currently two main molecular techniques are used: fluorescence *in situ* hybridization (FISH) or multiplex reverse transcription polymerase chain reaction (RT-PCR). FISH can be performed on interphase nuclei from cell suspensions, touch prints, or frozen or fixed paraffin-embedded sections most commonly using break-apart *PDGFB* or *COL1A1-PDGFB* fusion approach (Figure 2). On the other hand, RT-PCR requires RNA extracted from tumor fragments and necessitates the simultaneous use of several *COL1A1* primers (multiplex approach) as the breakpoint can randomly occur between exons 6 and 47 [43–45]. 

## 3. Targeted Therapy

**Figure 3:**
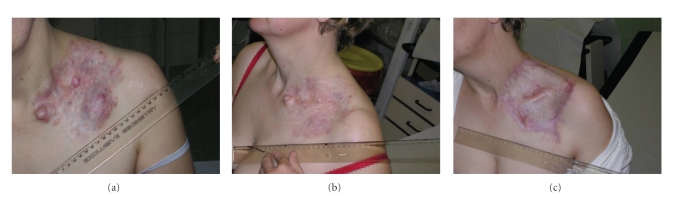
Images of advanced dermatofibrosarcoma protuberans of the supraclavicular region before and after therapy with imatinib, and after resection of residual disease. The patient is now 3 years free of disease.

Advances in the understanding of molecular mechanisms of DFSP resulted in the implementation of targeted therapy based on PDGFR inhibition to the treatment of this sarcoma. Imatinib mesylate is a tyrosine kinase inhibitor rationally developed and specifically directed against BCR/ABL, KIT, FMS (receptor for Colony Stimulating Factor 1), ARG (ABL-related gene), and PDGFR alpha and beta. It has been also found to be the first effective systemic therapy in DFSP. Imatinib competes with adenosine triphosphate (ATP) molecule, blocking tyrosine kinase receptor ability for autophosphorylation, which in return results in inhibition of the aberrant signal transduction pathway and partial restoration of proper intracellular signaling. The observation that autocrine overproduction of PDGFB caused by gene rearrangement is a key pathogenetic factor [33, 34] forced the *in vitro* research, which showed inhibition of DFSP cells growth *in vitro* after exposure to imatinib [36, 46]. The further demonstration of the imatinib inhibitory effect on six different DFSP cell lines both *in vitro *and *in vivo* [37] has led to the investigation of this new therapeutic approach in the clinic. Early case reports on small series of patients suggested the usefulness of imatinib in metastatic and locally advanced DFSP [47–52]. Next series of 10 patients with locally advanced and/or metastatic DFSP treated within Imatinib Target Exploration Consortium Study B2225 showed responses in all patients, including complete responses in five out of 10 of locally advanced cases and one partial response lasting seven months in metastatic case [53]. As a consequence imatinib was registered as a therapy of choice in advanced (inoperable and/or metastatic) DFSPs (Figure 3). In a phase II trial [54] evaluating the activity of imatinib in life-threatening malignancies expressing imatinib-sensitive tyrosine kinases DFSP was the only one of five tumor types in which a notable activity was shown including extensive regression in 10/20 cases (50% partial remissions, 33,3% complete remissions). 

Combined analysis of prematurely closed, two phase II, single arm, open-label trials on efficacy of imatinib in advanced DFSP (European Organisation for Research and Treatment of Cancer no. 62027 and the Southwest Oncology Group no. S0345) has demonstrated the clinical benefit with rate exceeding 70% and median time to progression of 1.7 years on 25 patients with advanced DFSP [55]. Although there were some differences in both trials' design, the observed responses' rates were similar. These results imply that the imatinib dose of 400 mg daily has similar efficacy to 800 mg daily in this entity. Rutkowski et al. [56] have proved striking activity of imatinib mesylate in advanced DFSP in the group of 15 patients treated with imatinib in routine clinical practice outside any trial, with clinical benefit rate approaching 80% as well as median PFS and OS being not reached. In Table 1 the efficacy results of imatinib in advanced DFSPs from pooled analysis of phase II trials [55] and 15 patients treated outside clinical trials is presented [56].

It has also been shown that DFSPs-FS with t(17;22) are still imatinibsensitive although responses seem to last shorter [57] while DFSPs-FS lacking the specific aberration do not respond to the treatment [53]. Therefore the confirmation of the molecular target (*COL1A1-PDGFB* fusion) presence seems to be obligatory in every case prior to the start of imatinib therapy. 

 Complete, wide surgical excision is the standard treatment in localized, resectable cases, and in advanced cases it may result in cosmetic disfigurement or serious functional impairment. Thus the neoadjuvant imatinib strategy leading to tumor downstaging and decrease of excision morbidity by tissue-sparing appears to be very attractive. Kérob et al. [58] presented report on 25 resectable DFSP (median size: 4.5 cm) treated in phase II trial with preoperative imatinib at the dose of 600 mg daily for two months. The objective partial response according to RECIST was observed in nine cases (36%). The median relative tumor volume decrease was 20% (range: 12.5–100%). Available clinical data indicate that some DFSP patients initially evaluated as unresectable/metastatic or necessitating mutilating surgery turned out to have resectable tumor after imatinib therapy. This rational approach enabling achievement of complete remission may be potentially curative, although longer followup is needed. Further studies are required for elucidating whether preoperative imatinib therapy reduces the need for wide surgical margins or whether imatinib has activity as adjuvant therapy in cases with positive margins after excision or in other high-risk patients. 

Majority of patients treated with imatinib experienced side effects during treatment, but almost all are mild and manageable. The most common were fluid retention/edema, anemia, fatigue, nausea, skin rash, thrombocytopenia, vomiting, neutropenia, and diarrhea, and they are similar to those observed in patients with gastrointestinal stromal tumor (GIST). 

There are still several questions regarding imatinib mechanism of action, and possible resistance to this targeted therapy in DFSP. There is also a need to identify novel predictive molecular markers for patients' outcome. It was presumed that imatinib effect resulted from inhibition of PDGFR phosphorylation. Surprisingly, clinical activity of imatinib in DFSP is striking even in DFSP expressing relatively low amounts of activated receptor. It seems that inhibition of low-level receptor tyrosine kinase may be effective clinically if tumor cells are dependent on that signaling mechanism, what has been observed also in pigmented villonodular synovitis/tenosynovial giant-cell tumor [59, 60]. The better understanding of the downstream effects caused by imatinib-PDGFB interaction would allow defining additional treatment strategies for DFSP patients. In case of disease progression after initial response to imatinib the investigation of other multitargeted tyrosine kinase inhibitors seems to be justified. 

To summarize, imatinib therapy is currently the gold standard in the treatment of inoperable and/or metastatic and/or recurrent cases of DFSP, and this targeted therapy may potentially facilitate resection or decrease possible disfigurement. Significant percentage of patients may be rendered free of disease by surgery of residual disease following partial imatinib responses. Current therapy of DFSP with t(17;22) translocation should be conducted by multidisciplinary team, including oncological surgeon. The use of imatinib mesylate as initial therapy should be considered to decrease possible extent of surgery and related morbidity.

## Figures and Tables

**Table 1:** The best overall responses, progression, and survival status in combined phase II clinical trials [55] and in group of patients treated outside clinical trials [56].

	Group of 24 patients treated in phase II trials [55]	Group of 15 patients treated outside clinical trials [56]
	*N* (%)	
Progression status		
Progression-free	12 (50)	11 (73)
Progression	12 (50)	4 (27)
Survival status		
Alive	18 (75)	12 (80)
Dead	6 (25)	3 (20)
Best overall response		
Partial response	11 (45.9)	11 (73)
Stable disease	6 (25)	1 (7)
Progressive disease	4 (16.6)	3 (20)
Not evaluable	3 (12.5)	0
